# Scouting for Naturally Low-Toxicity Wheat Genotypes by a Multidisciplinary Approach

**DOI:** 10.1038/s41598-018-36845-8

**Published:** 2019-02-07

**Authors:** Rosa Pilolli, Agata Gadaleta, Gianfranco Mamone, Domenica Nigro, Elisabetta De Angelis, Nicola Montemurro, Linda Monaci

**Affiliations:** 1 Institute of Sciences of Food Production, National Research Council of Italy (ISPA-CNR), via Giovanni Amendola, 122/O - 70126 Bari, Italy; 2 Department of Agricultural & Environmental Sciences, Università degli Studi di Bari Aldo Moro, via G. Amendola, 165/A – 70126 Bari, Italy; 3 Institute of Food Sciences, National Research Council of Italy (ISA-CNR), via Roma, 64 - 83100 Avellino, Italy; 4 Department of Soil, Plant & Food Sciences, Università degli Studi di Bari Aldo Moro, via G. Amendola 165/, A – 70126 Bari, Italy

## Abstract

Over the last years, great efforts have been devoted to develop effective gluten detoxification strategies with a consequent detrimental alteration of the technological properties as well. Obtaining low-gluten products without affecting the rheological properties of wheat could still be considered a new challenge to face. In this investigation, we presented a comprehensive characterization of durum wheat genotypes aimed at identifying low gluten ones, which combine the potential lower toxicity/immunogenicity with conserved yield and rheological properties to encompass the perspective usability for bread or pasta making. A preliminary profiling of gluten proteins was accomplished by immunoassay-based quantification and liquid chromatography coupled to UV detection, focusing on the gliadin fraction as main responsible for immunoreactivity in celiac disease patients. In addition, data on grain protein content, grain yield per spike, dry gluten and gluten index were collected in order to provide complementary information about productivity-related traits and quali-quantitative characteristics related to wheat nutritional value and its technological properties. The whole pool of data was statistically evaluated driving to the selection of a preferred list of candidate low-toxicity genotypes that were subjected to *in-vitro* simulated gastroduodenal digestion and untargeted HR-MS/MS peptide identification. Finally, an in-silico risk assessment of potential toxicity for celiac disease patients was performed according to the most recent guidance provided by EFSA.

## Introduction

In the last decade, the ingestion of wheat has been associated with clinical disorders, such as celiac disease (CD), wheat allergy (WA) and non-celiac gluten sensitivity (NCGS), which are becoming epidemiologically more and more relevant with an estimated global prevalence of about 5%^1^. The trigger factor eliciting CD and WA are largely investigated, while on the contrary the pathophysiology of NCGS is still poorly understood^2^. The only effective treatment for subjects affected by gluten-related disorders consist in gluten-free diet. In particular, for CD patients the limit of gluten in their diet must be rigorously lower than 20 ppm. The increasing demand for gluten-free products to be destined to vulnerable consumers together with the growing consumption of such products by non-celiac consumers, has caused a steadily increasing expansion of the gluten-free market in the last five years^1^. Moreover, the mainstream of a gluten-free diet in the general population has markedly increased in recent years. This increasing adoption of a gluten-free diet by people without celiac disease has occurred in conjunction with speculation that gluten may have a deleterious role in health outcomes even in the absence of gluten sensitivity^3,4^. However, evidence supporting gluten avoidance for physical symptoms or diseases not specifically related to gluten-mediated immunologic disease is not convincing^5–7^.

Over the last years, important efforts have been devoted to the development of technological approaches for wheat detoxification with successful results^8–11^. The sourdough fermentation showed the most promising results, also including a biotechnology strategy that allowed the complete gluten degradation prior to consumption^12^. The main achievements in the field were recently reviewed^13–15^, highlighting as main drawback the detrimental alteration of the technological properties as well.

The identification of wheat genotypes with reduced gluten content and having naturally low amounts of epitopes toxic for celiac patients was recently re-evaluated as option for new breeding strategies. The varietal selection undertaken by breeders in the last decades aimed at increasing productivity traits and improving rheological properties; as side effect it also caused a considerable impoverishment of the genetic diversity of wheat varieties present on the market^16^. Starting from this, the researchers encouraged a return to “old” wheat lines to be characterized in light of their potential to encode a lower number of celiac disease epitopes^17–21^. Indeed, wheat genotypes may differ significantly in the number and content of T-cell–stimulatory epitopes^22^. However, the genetic diversity itself cannot be used as a standalone approach for the development of celiac-safe wheat-based products; in fact, gluten levels below 20 ppm are required according to the Food and Drug Administration and Codex Alimentarius, which are not realistic for unprocessed wheat. Still, selecting varieties having naturally low amount of toxic epitopes can represent convenient bases for breeding practices and for the development of new detoxification strategies.

Several very recent investigations compared the proteomic profile of old and modern varieties, through various analytical approaches. The reported results often limited to a broad set of genotypes were very heterogeneous and sometimes contrasting, mainly because different methodological approaches were selected and direct comparison of the results is not always suitable. Predicting genotypes potential toxicity only by means of in silico or *in-vitro* experiments might be approximate and caution should be taken in drawing conclusions. A consensus about how to predict in silico and *in vitro* the genotype toxicity would be highly encouraged since an harmonization of the methods would improve the results comparability among different research group and independent investigations. Our personal opinion is that the EFSA guidance for allergenicity risk assessment in genetically modified plants^23^ provides very useful recommendations that should be followed in light of an harmonization of the methods.

So far, the characterization of wheat genotypes has been typically carried out according to two main strategies. The first aims at screening wide collections by combining proteomic and immunochemical analysis^24^; these investigations provided a general comparison of the proteomic profiles, focusing mostly on the expression level of gluten proteins^25–28^. Alternatively, in-depth investigations have been also proposed in the literature, which grounds on the evaluation of protein digestibility by *in-vitro* simulated human gastroduodenal digestion of wheat flours, and LC-MS/MS identification of resistant protein or peptides^29–31^. In the latter, information about the capability of immunogenic/toxic epitopes to reach intact the epithelium gut and trigger immune response can be retrieved. As advanced step, the immune stimulatory properties of digested samples can be estimated by T cell lines isolated from jejunal biopsis of CD patients. In a recent paper by Gianfrani *et al*. (2015) *Triticum monococcum* ancient cultivar, was analyzed and proposed as candidate low-toxic species for celiac disease patients. Authors demonstrated that the gliadins proteins from *T. monococcum* are sufficiently different from common hexaploid wheat with a lower number of resistant epitopes surviving the *in-vitro* simulated gastro-intestinal digestion^32^.

In this work, we presented the detailed characterization of a tetraploid wheat collection by a multidisciplinary approach. In addition to conventional proteomic profiling, focusing on the gliadin fraction, we also evaluated yield and quality traits in order to encompass the perspective usability of the wheat grains for bread or pasta making. Indeed durum wheat bread–making is an established tradition in southern Italy and in the Mediterranean countries^33,34^. The data gathered were critically discussed and statistically evaluated with the final aim to identify candidate genotypes displaying both a reduced gluten content and satisfactory rheological properties. These latter were subjected to *in-vitro* simulated gastroduodenal digestion and an in-silico risk assessment of potential toxicity for CD patients was carried out according to the most recent guidance provided by the European Food Safety Agency (EFSA).

## Results and Discussion

The focus of this investigation was placed on a set of 38 accessions of durum wheat (*Triticum turgidum)* selected from a wider collection of 240 genotypes, developed at University of Bari Aldo Moro, including both wild and cultivated accessions, which was already characterized in terms of genetic diversity and population structure^35^ and recently used for genome-wide association mapping of loci controlling yield components^36^. The considered accessions included both “cultivated” (C) and “non-cultivated” (NC) genotypes belonging to six subspecies (see Table 1 for details).

The first step was the identification of proper material references for comparative proteomic analysis. A commercial durum wheat semolina (REF 4) as mixture of modern common varieties was first considered. However, in order to widen the investigation we further characterized two well-known hexaploid accessions of *Triticum aestivum* (Chinese Spring and Spada, REF 1 and 2), as well as a diploid accession of *Triticum monococcum* which was recently proposed as low toxicity ancient genotype (REF 3)^31^.

### Characterization of the wheat collection

The selected tetraploid genotypes were first characterized by enzyme linked immunoassay (ELISA). Several sandwich or competitive ELISA kits are commercially available differing in the primary antibodies (monoclonal or polyclonal)^37–39^. Among these, R5 Mendez method, listed in the CODEX Alimentarius as a type 1 method for gluten detection in foods, relies on prolamin extraction by patented cocktail solution followed by R5 monoclonal antibody sandwich assay^40^. R5 antibody specifically recognizes the penta-peptide epitopes QQPFP, QQQFP, LQPFP, QLPFP, QLPYP, which are included into most of the celiac-toxic repetitive motifs able to induce the immune response in CD patients^41^. R5-ELISA kit directly quantifies the amount of wheat gliadins, but it recognizes also secalins and hordeins in rye and barley samples, respectively; according to the manufacturer protocol the gluten concentration is calculated by multiplying the gliadin concentration by 2. A linear correlation between R5-based assay and T-cell lines isolated from CD patients biopsies was previously demonstrated for a single genotype^42^. However, it deserves to be noted that the 5 AA motif recognized by the R5 antibody is much shorter that the 9 AA peptide binding register required to activate T-cells, therefore the R5-ELISA was performed to measure the gliadin content, and as main responsible of immunoreactivity in CD patients, to provide a preliminary estimate of potential differences in CD toxicity.

**Figure 1:**
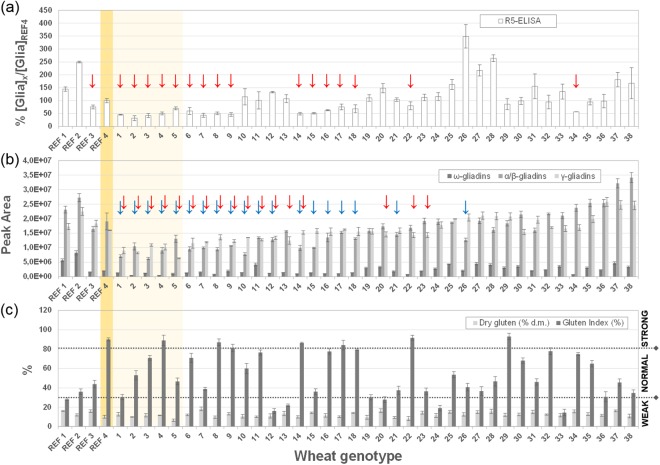
Comparison of R5-sandwich ELISA results and HPLC-UV relative gliadin quantification. Statistically significant differences at a significance level of 5% between commercial semolina and each genotype under investigation were determined by multiple t-tests and highlighted with arrows.

In order to provide a statistically relevant averaged result, two replicated extractions were carried out for each genotype and each extract was tested on two different wells of the same plate in different days. The coefficient of variation (CV %) was calculated as measure of the method reproducibility and found equal to 15% on average, which is totally acceptable for independent measurements based on immunological recognition^43^. Fitting the purpose of this investigation, the gliadin/gluten amount calculated by interpolation of calibration curves was indicated as percent ratio between the amount calculated for each genotype and the mean value calculated for the reference semolina (REF 4). Results are summarized in Fig. 1, panel a. The investigated wheat genotypes displayed a huge variability. The t-test statistical comparison of mean values at 95% confidence level highlighted a restricted list of genotypes, (marked with a red arrow in the Fig. 1a) with a relative amount of gliadin/gluten significantly lower than the reference semolina (REF 4).

Gliadins characterization by HPLC-UV analysis of the ethanol extracted fraction was carried out as previously reported^44^. The chromatographic peaks were assigned to ω-, α/β-, and γ- gliadins based on their retention times, and the peak areas were integrated in order to provide a relative quantitation of each gliadin type. Three independent extractions were performed for each genotype and averaged values with relevant standard deviations are shown in Fig. 1, panel b. As observed by ELISA, each class of gliadins presented a significant variability among different genotypes, and some of them, highlighted with arrows, presented a statistically lower expression of α/β- or γ-gliadins at 95% confidence level (blue and red arrows, respectively). We reported as Supplementary Information paired scatterplots, which highlighted a linear correlation between the R5-ELISA and the HPLC-UV analyses for most genotypes. As preliminary data, the experimental evidences about minor R5-reactivity and minor expression of α/β- or γ-gliadins suggested these as good candidates for lower gluten content genotypes endowed with lower toxicity/immunogenicity.

As following step, aiming at combining the potential lower toxicity/immunogenicity with reasonable grain yield and conserved rheological properties, some variables affecting the grain quality were investigated. In particular, the grain yield per spike (GYS), as productivity-related trait in wheat, the grain protein content (GPC), the dry gluten (DG) and the gluten index (GI), representing quali-quantitative characteristics related to wheat nutritional value and the related technological properties, were taken into consideration. The latter are important factors affecting pasta consistency and resistance to over-cooking^45^, as well as bread volume^46^. GI has been widely used for evaluating gluten strength (GI < 30% = weak; 30–80% = normal; >80% = strong)^47^, influenced both by environmental and genetic factors, and it has been reported to show high heritability^48^. GI instrumentally reproduces the manual gluten quality evaluation, thus avoiding any influence of the operator on the results.

GYS, GPC, DG and GI were experimentally determined for all the accessions including also the four reference samples (see Table 1), in five environments. The GYS value was not available for REF4 sample, as commercial product, purchased by the market. Average GYS across the five environments ranged from 1.00 g (cv Chinese Spring, sample REF 1) to 2.70 g (cv Cappelli, sample 7), with a mean value among the genotypes in each environment being 2.08 g. In addition, GPC values (expressed in % on dry weight) showed high variability: averages ranged from 12.6% (sample REF 4) to 19.4% (MG4330/66), with a mean value of 14.3%. Dry gluten (DG) and gluten index (GI) were analyzed on 10 g of milled grain samples, the experimental values were displayed in Fig. 1, panel c. DG showed values ranging from 6.5 to 18% d.m., with a mean value of 12.5% d.m. As for the gluten strength, the collection presented a high variability for GI, with averaged values ranging from 15% to 93%. According to the previously reported classification, most of the considered genotypes showed a normal GI, while six genotypes were classified as weak gluten strength and seven as strong gluten index (see Table 1). The very low GI values (samples 12, 24, 33) indicated a very weak and sticky gluten, which would be insufficient for both bread –making and pasta production^49^, whereas the very high GI ≥ 90% (samples 4, 8, 22, 29) indicated a strong gluten network with optimal pasta-making properties^50^. As for bread making, the majority of the grains tested providing GI ≥ 55% would be able to provide good bread making performances, according to Har Gil *et al*.^51^.

**Table 1:** Detailed list of *Triticum* accessions included in the wheat collection (Grouping: C = cultivated, NC = non cultivated) and summary of the grain quality features: grain yield per spike (GYS), grain protein content (GPC), dry gluten (DG), gluten Index (GI).

Sample code	Accession	Taxonomic classification	Year of release	C/NC	Origine	GYS (g)	GPC (% d.m.)	DG (% d.m.)	GI (%)
REF 1	Chinese Spring	*T. aestivum ssp. aestivum*		C	—	1.0 ± 0.2	14.3 ± 1.0	16.0 ± 0.6	*28.1 ± 1.3
REF 2	Spada	*T. aestivum ssp. aestivum*		C	—	1.6 ± 0.7	12.7 ± 0.9	12.0 ± 1.1	**36 ± 3
REF 3	ID3	*T. monococcum ssp. monococcum*	—	NC	—	1.2 ± 0.7	16.9 ± 1.9	16.0 ± 1.4	**44 ± 4
REF4	Commercial Semolina	—	—	—	—	NA	12.6 ± 0.4	10.1 ± 1.5	***90.0 ± 1.6
1	Duetto	*T. turgidum ssp. durum*	2002	C	Italy	2.4 ± 0.3	14.3 ± 1.3	12.8 ± 1.9	**30 ± 3
2	Colosseo	*T. turgidum ssp. durum*	1995	C	Italy	2.4 ± 0.2	13.7 ± 1.1	9.9 ± 0.5	**53 ± 5
3	Lloyd	*T. turgidum ssp. durum*	1983	C	United States	2.3 ± 0.4	13.3 ± 1.8	11.7 ± 1.7	**71 ± 2
4	Neolatino	*T. turgidum ssp. durum*	2007	C	Italy	2.5 ± 0.2	13.3 ± 0.7	11.6 ± 0.3	**89 ± 5
5	PI 56263	*T. turgidum ssp. turgidum*	—	NC	Portugal, Lisboa	2.6 ± 0.5	15.0 ± 1.8	6.5 ± 1.2	**47 ± 3
6	Ciccio	*T. turgidum ssp. durum*	1996	C	Italy	2.3 ± 0.3	13.3 ± 0.6	12.1 ± 0.9	**71 ± 5
7	Cappelli	*T. turgidum ssp. durum*	1915	C	Italy	2.70 ± 0.13	16.1 ± 1.2	18 ± 2	**38.8 ± 1.7
8	Iride	*T. turgidum ssp. durum*	1996	C	Italy	2.5 ± 0.2	13.0 ± 0.9	9.7 ± 1.3	**87 ± 3
9	Nefer	*T. turgidum ssp. durum*	1996	C	France	1.9 ± 0.3	14.7 ± 1.3	13.2 ± 1.2	**81 ± 4
10	Provenzal	*T. turgidum ssp. durum*	1998	C	Italy	2.0 ± 0.3	14.0 ± 1.6	10.7 ± 1.9	**60 ± 5
11	Kiperounda	*T. turgidum ssp. durum*	1956	C	Marocco	2.0 ± 0.4	15.0 ± 0.9	10.2 ± 0.8	**76 ± 3
12	PI 352542	*T. turgidum ssp. turgidum*	—	NC	France	2.3 ± 0.9	14.0 ± 1.0	11 ± 2	*16 ± 3
13	Russello	*T. turgidum ssp. durum*	1928	C	Italy	2.1 ± 0.7	15.3 ± 1.7	14 ± 2	*22.0 ± 1.3
14	Canyon	*T. turgidum ssp. durum*	2005	C	Italy	2.20 ± 0.15	13.2 ± 0.7	10.0 ± 1.2	**86.3 ± 0.9
15	Capeiti-8	*T. turgidum ssp. durum*	1940	C	Italy	1.97 ± 0.19	14.3 ± 1.1	14.2 ± 0.8	**36 ± 3
16	Duilio	*T. turgidum ssp. durum*	1984	C	Italy	2.2 ± 0.3	13.3 ± 0.9	11.5 ± 1.9	**78 ± 3
17	Gianni	*T. turgidum ssp. durum*	1992	C	Italy	2.29 ± 0.11	12.9 ± 0.9	10.2 ± 0.8	**84 ± 5
18	Athena	*T. turgidum ssp. durum*	1982	C	Italy	2.1 ± 0.4	15.1 ± 1.0	14.1 ± 0.6	**79.4 ± 0.8
19	PI 167481	*T. turgidum ssp. turanicum*	—	NC	Turkey, Denizli	1.9 ± 0.3	13.9 ± 1.2	10 ± 2	**30 ± 3
20	MG4328/61	*T. turgidum ssp. dicoccoides*	—	NC	—	1.3 ± 0.3	17 ± 2	16 ± 2	*28 ± 3
21	PI 387479	*T. turgidum ssp. polonicum*	—	NC	Ethiopia	1.8 ± 0.3	14.4 ± 1.0	9.3 ± 0.9	**37 ± 4
22	Saragolla	*T. turgidum ssp. durum*	2004	C	Italy	2.5 ± 0.4	13.9 ± 0.8	8 ± 2	***92 ± 3
23	Isa	*T. turgidum ssp. durum*	1975	C	Italy	2.1 ± 0.3	13.9 ± 0.5	14.4 ± 1.6	**36 ± 3
24	Latino	*T. turgidum ssp. durum*	1982	C	Italy	2.5 ± 0.3	13.1 ± 0.7	11.5 ± 2	*19 ± 3
25	PI 278350	*T. turgidum ssp. turanicum*	—	NC	Italy	2.3 ± 0.3	13.6 ± 0.4	15.2 ± 1.4	**54 ± 3
26	PI 192658	*T. turgidum ssp. turanicum*	—	NC	Morocco	2.4 ± 0.4	14.6 ± 0.6	12.6 ± 1.7	**41 ± 4
27	MG 4387	*T. turgidum ssp. dicoccum*	—	NC	United kingdom	1.8 ± 0.3	16.0 ± 1.9	15.0 ± 1.4	**37 ± 4
28	PI 352323	*T. turgidum ssp. dicoccoides*	—	NC	Asia Minor	1.1 ± 0.3	18.2 ± 1.9	16 ± 2	**47 ± 5
29	Cirillo	*T. turgidum ssp. durum*	1992	C	Italy	2.2 ± 0.4	13.7 ± 1.9	12.1 ± 1.7	***93 ± 3
30	Grecale	*T. turgidum ssp. durum*	2002	C	Italy	2.3 ± 0.5	13.1 ± 0.8	12.6 ± 1.4	**68 ± 3
31	Valnova	*T. turgidum ssp. durum*	1975	C	Italy	2.5 ± 0.4	14.0 ± 0.9	15.1 ± 1.7	**46 ± 3
32	Alemanno	*T. turgidum ssp. durum*	2006	C	Italy	2.49 ± 0.09	12.8 ± 0.8	12.4 ± 0.6	**78 ± 3
33	PI 157983	*T. turgidum ssp. turgidum*	—	NC	Italy, Sicily	2.5 ± 0.5	13.8 ± 0.5	11.8 ± 1.7	*15 ± 3
34	Creso	*T. turgidum ssp. durum*	1974	C	Italy	2.2 ± 0.3	13.4 ± 1.3	15.6 ± 1.6	**74.8 ± 1.5
35	Appio	*T. turgidum ssp. durum*	1982	C	Italy	2.3 ± 0.4	12.7 ± 1.1	12.9 ± 1.4	**65 ± 3
36	Enduro	*T. turgidum ssp. durum*	1991	C	Italy	2.2 ± 0.3	13.3 ± 0.8	11.6 ± 1.1	**31 ± 5
37	MG4330/66	*T. turgidum ssp. dicoccoides*	—	NC	—	1.2 ± 0.3	19.4 ± 1.5	16.2 ± 0.9	**46 ± 4
38	PI 221423	*T. turgidum ssp. turgidum*	—	NC	Portugal	2.68 ± 0.14	14.5 ± 1.3	10.9 ± 1.9	**35 ± 3

*Weak gluten strength GI < 30%; **normal gluten strength GI 30–80%; ***strong gluten strength GI > 80%.

Aiming at improving the understanding and the significance of the collected results, we performed a statistical evaluation of the multivariate data system describing the whole collection. Pearson’s product moment correlations between qualitative and quantitative grain features were reported in Table 2, with relevant p-values for statistical significance. Since both R5-reactivity and single gliadins quantification by HPLC-UV are strictly related to the abundance of gliadin proteins in the genotypes, a high correlation among these features was expected and lastly confirmed by the experimental data. Significant negative correlations were found between GYS and ω-gliadins (expression level and percentage weight on total gliadins), as well as GYS and GPC (R = −0.609 with p < 0.001). The negative correlation with GPC was expected, and confirmed by a number of studies. GPC is a typical quantitative trait, controlled by several genes and strongly affected by environmental factors. During the last 2 decades, breeding programs have been focusing on its improvement, but this has been limited due to the negative correlation between yield and GPC found at different level, with correlations ranging from −0.20 to −0.80^52^. Several hypothesis have been formulated to explain this negative correlation, including environmental factors, genetic components^53^, dilution of grain nitrogen with a much larger grain biomass accumulation^54,55^, or bio-energetic requirements for synthesis of carbohydrates and proteins^56^. GYS was also found to negatively correlate with R5-ELISA (R = −0.342 with p < 0.05) and DG (R = −0.448 with p < 0.01), respectively. Both these experimental evidences were expected for the same reasons of the negative correlation with GPC since the two variables R5-ELISA and DG both depend on the absolute gluten content, which represents about 80% of the total grain protein. As a confirmation both R5-ELISA and DG featured a positive correlation with GPC (R = 0.338 with p < 0.05, and R = 0.513 with p < 0.001, respectively). In addition, negative correlation between yield components and DG was also reported by other authors^49^.

**Table 2:** Pearson correlation coefficients (R) among all the investigated variables with relevant p-values calculated with n = 41 (ns = not significant, p > 0.05).

	α/β-glia	γ-glia	TOT-glia	% ω-glia	% α/β-glia	% γ-glia	R5-ELISA	GYS	GPC	GI	DG
ω-glia	**0.628**	**0.587**	**0.727**	**0.879**	0.077^ns^	**−0.459**	**0.642**	**−0.499**	0.212^ns^	**−0.340**	0.302^ns^
	*p* < *0.001*	*p* < *0.001*	*p* < *0.001*	*p* < *0.001*	*p* = *0.631*	*p* = *0.003*	*p* < *0.001*	*p* < *0.001*	*p* = *0.184*	*p* = *0.030*	*p* = *0.055*
α/β-glia		**0.766**	**0.956**	0.267^ns^	**0.575**	**−0.666**	**0.427**	−0.217^ns^	0.130^ns^	−0.261^ns^	0.226^ns^
		*p* < *0.001*	*p* < *0.001*	*p* = *0.092*	*p* < *0.001*	*p* < *0.001*	*p* = *0.005*	*p* = *0.174*	*p* = *0.416*	*p* = *0.100*	*p* = *0.155*
γ-glia			**0.907**	0.256^ns^	−0.020^ns^	−0.093^ns^	**0.618**	−0.298^ns^	0.176^ns^	−0.192^ns^	**0.334**
			*p* < *0.001*	*p* = *0.105*	*p* = *0.900*	*p* = *0.563*	*p* < *0.001*	*p* = *0.058*	*p* = *0.272*	*p* = *0.229*	*p* = *0.033*
TOT-glia				**0.376**	**0.331**	**−0.481**	**0.574**	**−0.310**	0.172^ns^	−0.271^ns^	0.301^ns^
				*p* = *0.015*	*p* = *0.034*	*p* = *0.001*	*p* < *0.001*	*p* = *0.049*	*p* = *0.281*	*p* = *0.086*	*p* = *0.056*
% ω-glia					−0.124^ns^	**−0.321**	**0.499**	**−0.470**	0.236^ns^	**−0.322**	0.263^ns^
					*p* = *0.442*	*p* = *0.041*	*p* < *0.001*	*p* = *0.002*	*p* = *0.138*	*p* = *0.040*	*p* = *0.096*
% α/β-glia						**−0.900**	−0.097^ns^	0.112^ns^	−0.012^ns^	−0.140^ns^	−0.127^ns^
						*p* < *0.001*	*p* = *0.547*	*p* = *0.486*	*p* = *0.942*	*p* = *0.383*	*p* = *0.428*
% γ-glia							−0.127^ns^	0.099^ns^	−0.092^ns^	0.275^ns^	0.006^ns^
							*p* = *0.430*	*p* = *0.537*	*p* = *0.567*	*p* = *0.082*	*p* = *0.971*
R5-ELISA								**−0.342**	**0.338**	**−0.421**	0.241^ns^
								*p* = *0.029*	*p* = *0.031*	*p* = *0.006*	*p* = *0.129*
GYS									**−0.609**	0.222^ns^	**−0.448**
									*p* < *0.001*	*p* = *0.162*	*p* = *0.003*
GPC										−0.301^ns^	**0.513**
										*p* = *0.056*	*p* < *0.001*
GI											−0.257^ns^
											*p* = *0.105*

Significant R values were highlighted in bold font style.

Interestingly, gluten index was found to negatively correlate to R5-ELISA (R = −0.421 with p < 0.01) meaning that within the investigated collection, high gliadin content decreases the gluten strength. This trend is strictly related to the composition of the HMW glutenins as reported by Van den Broeck *et al*.^57^. In general, our results agree with those previously documented by De Santis *et al*.^25^, and De Vita *et al*.^58^, which also reported improvement in gluten strength in the modern genotypes. In our material, the highest values of GI were reported in cultivars released in the last 25 years, confirming that durum wheat breeding programs determined improvements in pasta-making quality without any increment of toxic epitopes related to celiac disease. Similar conclusions were reported for breeding programs in United States by Kasarda^59^.

Given the high correlation observed among the different variables, principal component analysis (PCA) was performed based on the iterative NIPALS algorithm, in order to reduce the complexity of the multivariate system and to highlight potential trends of the whole data pool. The significance of the PC fitting model was evaluated using the method of v-fold cross-validation which allowed the extraction of the first five PCs as significant (see Table 3), featuring a cumulative explained variation (R^2^X(cum)) of 89.7% and predicted variation of 99.8% (Q^2^(cum)).

**Table 3:** Summary of the Principal Component Analysis based on NIPALS algorithm calculation performed with Statistica 7.0 software.

PC	R^2^X	R^2^X (Cum.)	Eigenvalues	Q^2^	Q^2^ (Cum.)
1	0.4216	0.4216	5.05	0.3690	0.3690
2	0.1958	0.6173	2.35	0.5699	0.7286
3	0.1166	0.7340	1.40	0.7130	0.9221
4	0.0941	0.8280	1.13	0.7976	0.9842
5	0.0687	0.8968	0.83	0.8860	0.9982

Diagnostics on both the observation and the variable levels was accomplished without further assumption, in order to highlight potential data outliers, variables reciprocal relation and individual contribution to the PC model. Neither strong outliers by Hotelling T^2^ control chart, neither moderate outliers by SPE (square of predictions error) chart were detected in the data set, the upper control limit being set at 99%. The importance of the variables resulted always higher than 0.67. We already discussed about variables correlation, further diagnostics on their contribution to the PC was accomplished by critical evaluation of the loading values. The specific gliadin amounts (ω-, α/β-, γ-gliadins), the total gliadin, and the R5-reactivity mainly contributed to the PC 1 (loadings >0.71); the % ω-gliadin equally influenced the PC1 and PC4 (loadings 0.66 and 0.63 respectively); the % α/β- and % γ-gliadins considerably contributed to the PC 2 (loadings >0.70); the gluten index mainly contributed to PC5 (loading 0.73) and moderately to PC1 (loading −0.47), whereas GPC moderately influenced almost equally the first three principal components (loading values ranging from 0.42 to 0.52); similarly DG influenced almost equally PC1, PC2 and PC4 (loading values ranging from 0.44 to 0.46) and the GYS contributed to the first two PCs (loading values ranging from 0.46 to 0.55).

**Figure 2:**
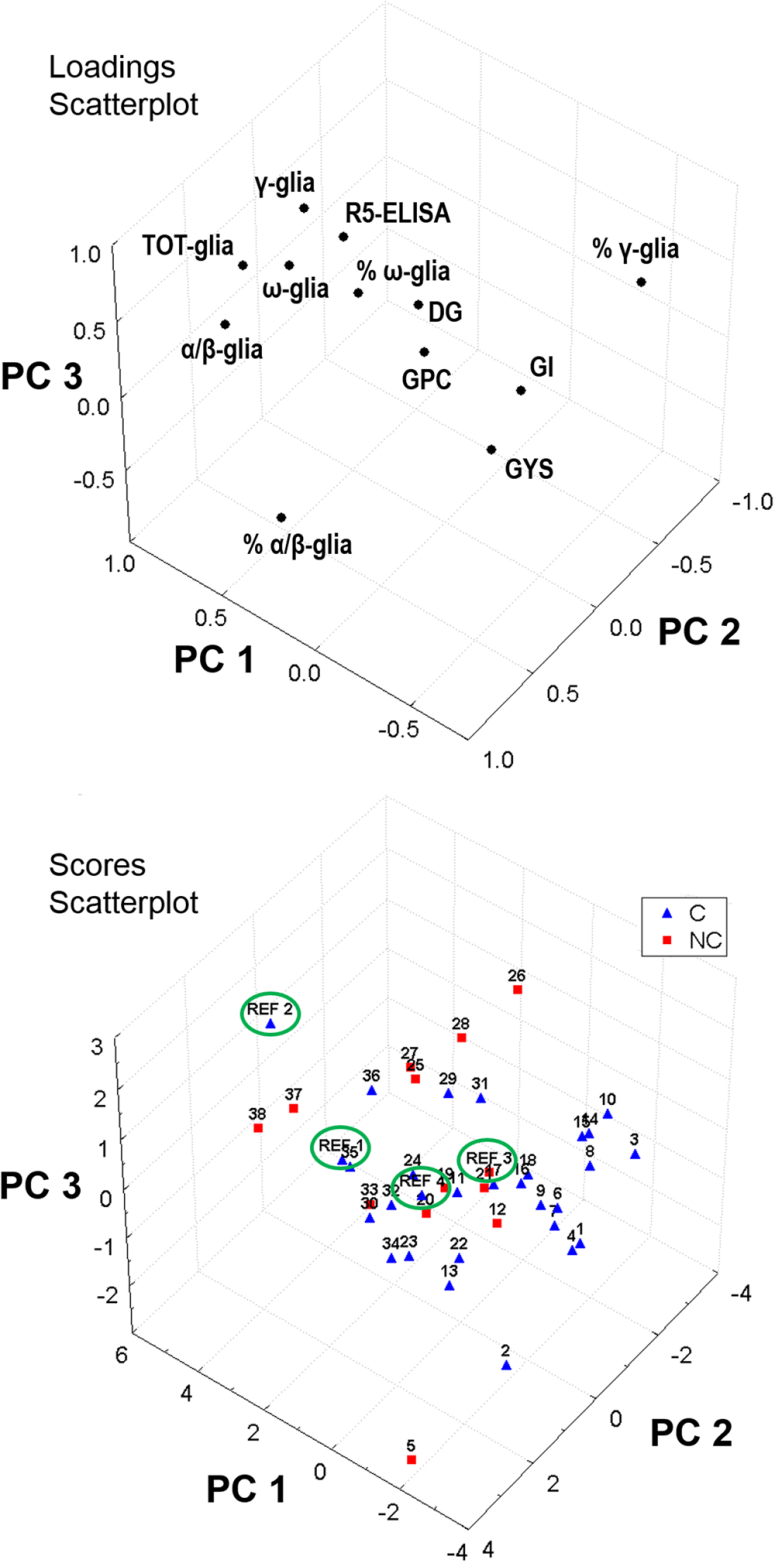
Loadings and Scores plots for the first three components obtained by Principal Component Analysis on 12 variables and 42 cases.

The scatterplots of loadings and scores for the first three components were displayed in Fig. 2 for visual inspection of the reduced dimension data pool. The spatial distribution of the genotypes did not allow a clear separation between cultivated (C) and non-cultivated (NC) wheat varieties, and also the two hexaploid accessions (REF 1 and REF 2, in the figure) and the diploid accession (labelled as REF 3), which have a different genetic makeup, did not segregate apart. The reference semolina sample (REF 4) lied in the middle of the pool.

**Figure 3:**
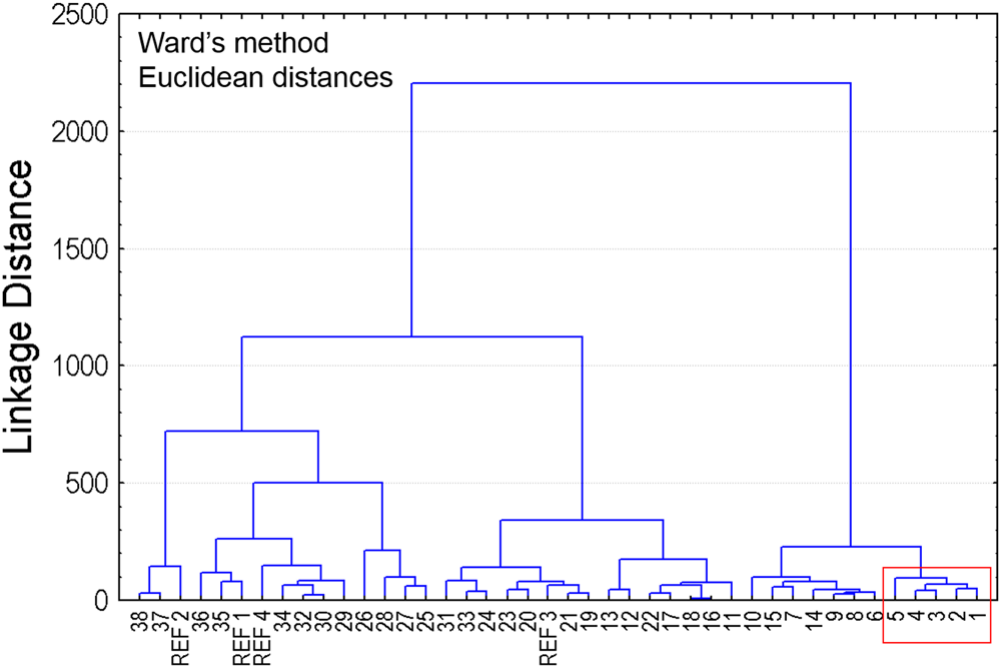
Hierarchical cluster analysis by joining tree was performed on original variables setting Ward’s methods amalgamation rule and selecting Euclidean distances.

In addition, a hierarchical cluster analysis (HCA) by joining tree was performed on original variables; given the higher unit scale of the HPLC-UV peak areas, the latter were scaled down to avoid over-weighting of this variable on the cluster calculation. The HCA was performed by setting Ward’s method as amalgamation rule and selecting Euclidean distances; the resulting dendrogram was reported in Fig. 3.

The HCA provided a dendrogram clearly characterized by three main clusters, which supported the proper selection of genotypes for further investigation. In particular, the focus was placed on the first five samples (labelled from 1 to 5), belonged to the same cluster, featuring the main distance from the references containing clusters. Given the significant correlations among the acquired variables, we also tried to perform HCA on a subset of selected variables. In particular, we removed the information of single gliadins types (α/β- glia, γ-glia, ω-glia) and of R5-reactivity, first and after also the % ω-glia, % α/β-glia, % γ-glia because the TOT-glia variable was significantly correlated with all those variables (data not shown). Reducing the number of variables resulted only in slight differences in the dendrograms, with the common finding that the first five genotypes belonged to the same cluster which is also different from the references’ ones. Scouting the raw data for these samples, it was noted that such genotypes featured all significantly lower R5-reactivity, together with lower α/β-, and γ- gliadins expression level than the semolina reference, whereas they displayed quite variable dry gluten % (from 6.5% to 12.8%) and gluten index % (from 30% to 89%). Indeed, despite the low gliadin content, the selected genotypes presented a heterogeneity in the gluten quality parameters fostering satisfactory technological properties for actual usability in wheat based foods.

### Evaluation of gastroduodenal digestibility and potential toxicity of selected genotypes

Data obtained from ELISA and HPLC analysis led to select five genotypes as potential candidate, that were further submitted to proteomic characterization (sample 1–5) before undertaking *in-vitro* assessment of their immunological properties. Considering the various approaches available in literature, we proceeded according to the most recent guidance provided by the European Food Safety Agency (EFSA)^23^, referring to allergenicity risk assessment of genetically modified plants. Such guidance addresses three main topics, two of them were useful for our case study, namely,(i) non-IgE mediated adverse immune reaction to foods and (ii) *in-vitro* protein digestibility tests. For the first topic, EFSA issued opinions to determine the safety profile of protein/peptide with regard to its potential to cause celiac disease. As for the *in-vitro* protein digestibility tests the panel did not provide a final guidance since an interim evaluation phase of the *in-vitro* gastroduodenal digestion (GDD) experiment is required. Therefore, we only shared the general statement on GDD data interpretation, concerning the lower limit of 9 amino acids (AA) in length, for peptide fragments persistent at GDD end-point, to be able to elicit immune-adverse reaction.

**Figure 4:**
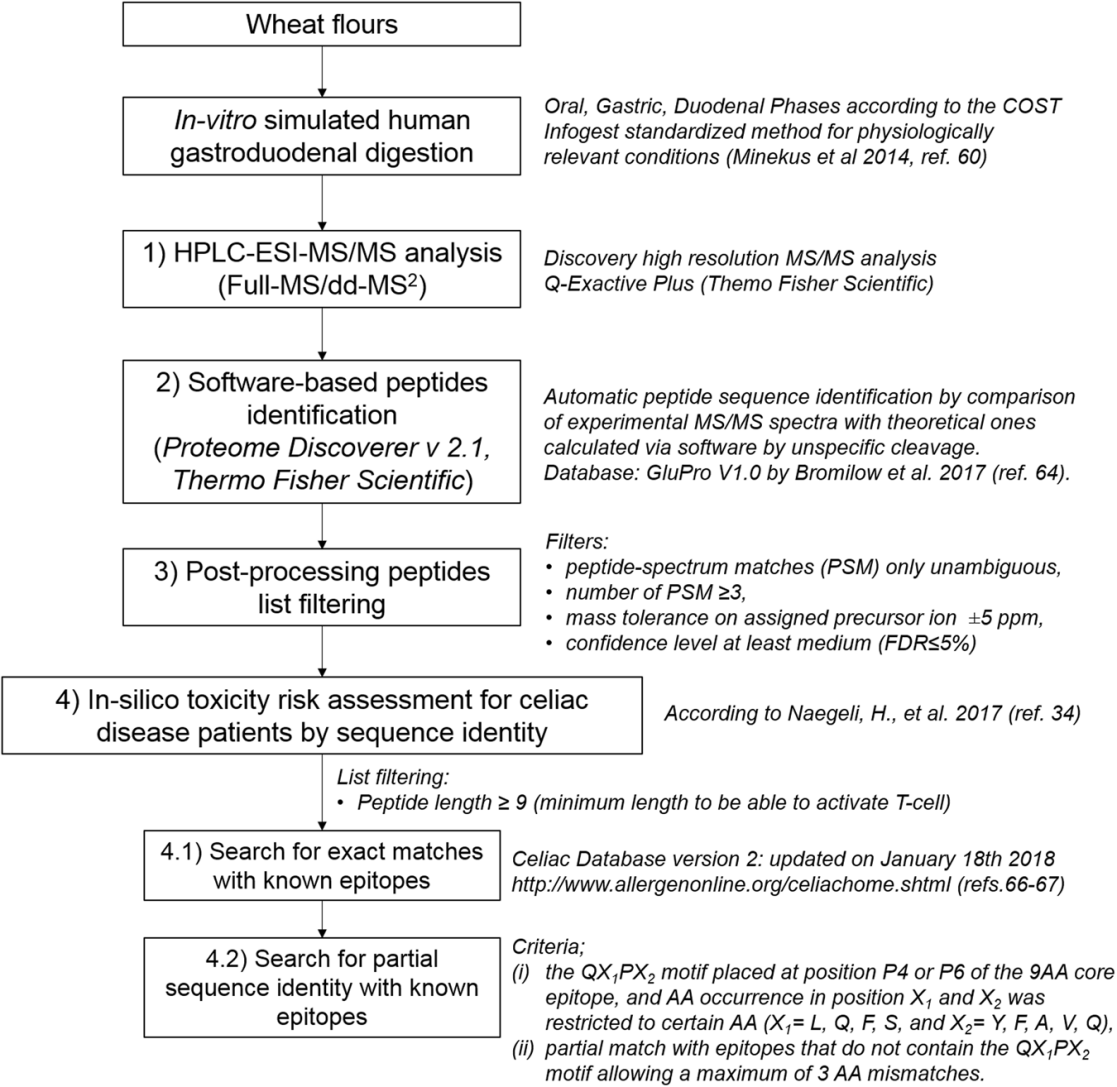
Workflow of the analytical strategy carried out for the identification by untargeted HR-MS/MS analysis of GD resistant peptides and in-silico toxicity risk assessment for celiac disease patients.

Starting from this background, *in-vitro* simulated human GDDs were carried out on raw flours of the five selected wheat genotypes (sample 1–5), also including semolina reference sample (REF 4). The full analytical workflow was summarized in Fig. 4. The standardized static method proposed within the COST Infogest network and cited in the guidance, was applied for physiologically relevant digestion conditions, in light of a perspective results comparability by method harmonization^60^. The protocol set parameters for the three phases of oral, gastric and duodenal digestion and was applied in the present study. Only the analytical readout of duodenal endpoint (2 h incubation) was analysed by untargeted high-resolution mass spectrometry after solid phase extraction (C18-based purification). The instrumental method was tailored to the comprehensive data acquisition with a wide acquisition range and a high tolerance for MS/MS event activation. The raw data were processed by the commercial software Proteome Discoverer 2.1 for protein/peptide sequence identification. Sequest HT searching algorithm against a customized database was applied. However, it deserves to be noted that here the peptide identification is complicated by the availability of full-length protein sequences from on-line databases and by the heterogeneity of the peptide mixture generated with not predictable specificity^31^. Given the high complexity of the enzyme mixtures used for GDD an ‘unspecific cleavage’ was set for peptide identification together with a wide range in the expected peptide lengths and molecular weight^61,62^. Such comprehensive set up for peptide searching would have raised exponentially the amount of data processing; therefore, in order to constrain the final software output to only the information fitting our purposes, we selected a customized restricted protein database, for specific searches. This approach was proved to provide better identifications compared to the use of large databases^63,64^.

Indeed, we uploaded and indexed the manually curated GluPro V1.0 database released as open-source by Bromilow *et al*. in 2017 comprising discrete unique full-length protein sequences representative of the different types of gliadin and glutenin components found in gluten^64^. Specific filters were also applied to select only peptide identified at the highest confidence level (see the methods section for details) and the total number of identified peptides for each sample was summarized in Table 4. Like already mentioned, peptides shorter than 9 AA cannot stimulate an immune response, since they would be unable to bind to MHC class II molecules and to activate T-cells (length lower than the peptide binding register^65^); therefore, a size cut-off of 9 AA was applied to investigate only peptide sequences resistant to GDD with T-cell stimulatory potential. Such restricted peptide list was screened for sequence identity with known T-cell stimulatory epitopes following the stepwise approach suggested by the EFSA guidance: (i) exact matches with known epitopes, (ii) partial sequence identity with case-by-case critical evaluation of number, type and position of AA mismatches.

**Table 4:** Summary of the GD peptides identified by untargeted HR-MS/MS analysis and checked for in-silico toxicity assessment (*after applying the following filters: peptide-spectrum matches (PSMs) only unambiguous, number of PSMs ≥ 3, mass tolerance precursor ion ≤5 ppm, confidence level at least medium).

SAMPLES	REF 4	1	2	3	4	5
Total identified peptides*	411	290	286	275	281	283
N° peptides with sequence length ≥9 AA	155	77	74	82	76	79
N° hazard peptides with 100% sequence identity with T-cell stimulatory epitopes	13	4	3	4	5	3
N° concerning peptides with partial match (QX_1_PX_2_ motif present in P4 or P6)	30	9	11	12	8	15
N° concerning peptides with partial match (QX_1_PX_2_ motif absent)	10	7	8	8	4	4

The first step was accomplished with the aid of on-line available bioinformatics tools. The CELIAC Database v.2 and the relevant tool for protein risk assessment was used to identify exact matches and substrings between the detected GDD resistant peptides and naturally occurring epitopes associated with CD^66,67^. In Table 4 we showed the results of this qualitative investigation reporting the total number of identified GD resistant peptides, and the number of peptides recognized as a safety hazard for full match with T-cell stimulatory epitopes. The absolute quantitation of such epitopes was out of the scope of this investigation. In addition, in Table 5 we also detailed some information about the experimental peptide-epitope matching (experimental m/z, peptide AA sequence, and protein belonging to) as well as the number of overlapping epitopes (n), their identification number (ID, like reported in Celiac Disease database^66^) and AA sequence, and the relevant restricted 9 AA core epitope (according to the current nomenclature proposed by Sollid *et al*.^68^. For the semolina sample (REF 4) we identified 13 resistant peptides encrypting full length stimulatory epitopes belonging to α/β-gliadins, γ-gliadins, ω-gliadins and LMW glutenins. According to previous investigations carried out by Prandi *et al*.^30^, the complexity of the model used to simulate the human GDD can influence the peptides pool detected by LC-MS/MS analysis. In particular, simplified protocols promoted the detection of α/β-gliadins whereas, the use of a complex digestion model simulating physiological conditions, fosters the proteolysis of γ-gliadins due to not only a better extractability of these proteins in presence of the digestive juices but also to a better bioaccessibility obtained with the more complex enzyme composition^29,30^.

**Table 5:** List of the GD resistant peptides identified by untargeted HR-MS/MS analysis and assessed to encrypt full length T-cell epitopes (*information retrieved by http://www.allergenonline.org/celiacbrowse.shtml^66^, **core epitope nomenclature by Sollid *et al*.^68^).

Sample	Peptides matching full length T-cell epitopes (100% sequence identity)			Known T-cell stimulatory epitopes			
	Experimental m/z [Da]	Sequence	Protein	n°*	Sequence (ID)*	HLA-DQ molecule*	9 AA Restricted epitope**
**REF 4**	1075,022 (+2)	QQ**PFPQTQQPQQPFPQ**QP	γ-gliadin	3	QQPQQPFPQ (472)	DQ2.5/DQ8	DQ2.5-glia-γ4c/DQ8-glia-γ1a
					PFPQTQQPQQPFPQ (553)	DQ8 (DQ2/8)	DQ2.5-glia-γ4c/DQ8-glia-γ1a
					PQTQQPQQPFPQ (926)	DQ2	DQ2.5-glia-γ4c/DQ8-glia-γ1a
	635,660 (+3)	Q**PFPQQPQQPF**PLQPQ	ω-gliadin	2	PFPQQPQQPF (538)	DQ2 (Glia-γ2 (p89-p102))	—
					FPQQPQQPF (542)	DQ2 (p90-p102)	—
	1409,676 (+1)	S**QQPQQPFPQ**PQ	γ-gliadin	1	QQPQQPFPQ (472)	DQ2.5/DQ8	DQ2.5-glia-γ4c/DQ8-glia-γ1a
	1419,705 (+1)	P**QQPQQPFPQ**PQ	γ-gliadin/ω-gliadin	1	QQPQQPFPQ (472)	DQ2.5/DQ8	DQ2.5-glia-γ4c/DQ8-glia-γ1a
	661,829 (+2)	QP**QQPFPQQPQ**	γ-gliadin/ω-gliadin	1	QQPFPQQPQ (438)	DQ2.5	DQ2.5-glia-γ5
	597,799 (+2)	P**QQPQQPFPQ**	γ-gliadin/ω-gliadin	1	QQPQQPFPQ (472)	DQ2.5/DQ8	DQ2.5-glia-γ4c/DQ8-glia-γ1a
	1198,585 (+1)	T**QQPQQPFPQ**	γ-gliadin	1	QQPQQPFPQ (472)	DQ2.5/DQ8	DQ2.5-glia-γ4c/DQ8-glia-γ1a
	549,272 (+2)	**QQPQQPFPQ**	γ-gliadin/ω-gliadin	1	QQPQQPFPQ (472)	DQ2.5/DQ8	DQ2.5-glia-γ4c/DQ8-glia-γ1a
	911,126 (+3)	PQ**QPFPQPQLPYSQ**PQPFRPQQP	α-gliadin	4	QPFPQPQLPY (42)	DQ2	DQ2.5-glia-α1a
					PFPQPQLPY (53)	DQ2.5	DQ2.5-glia-α1a
					QPFPQPQLPYSQ (164)	DQ2	DQ2.5-glia-α1a
					PFPQPQLPYSQ (166)	DQ2	DQ2.5-glia-α1a
	1253,631 (+2)	PQ**QPFPQPQLPYSQ**PQPFRPQ	α-gliadin	4	QPFPQPQLPY (42)	DQ2	DQ2.5-glia-α1a
					PFPQPQLPY (53)	DQ2.5	DQ2.5-glia-α1a
					QPFPQPQLPYSQ (164)	DQ2	DQ2.5-glia-α1a
					PFPQPQLPYSQ (166)	DQ2	DQ2.5-glia-α1a
	641,984 (+3)	QPQP**FRPQQPYPQ**SQP	α/β-gliadin	1	FRPQQPYPQ (93)	DQ2.5	DQ2.5-glia-α3
	541,937 (+3)	QQP**PFSQQQQPV**LP	LMW glutenin/γ-gliadin	1	PFSQQQQPV (706)	DQ2.2	DQ2.2-glut-L1
	774,383 (+2)	**PQQPFPQQPQQP**Q	ω-gliadin	3	PQQPFPQQPQQP (195)	DQ2	DQ2.5-glia-γ5
					PQQPFPQQPQQ (432)	DQ2	DQ2.5-glia-γ5
					QQPFPQQPQ (438)	DQ2.5	DQ2.5-glia-γ5
**1**	597,799 (+2)	P**QQPQQPFPQ**	γ-gliadin/ω-gliadin	1	QQPQQPFPQ (472)	DQ2.5/DQ8	DQ2.5-glia-γ4c/DQ8-glia-γ1a
	1198,584 (+1)	T**QQPQQPFPQ**	γ-gliadin	1	QQPQQPFPQ (472)	DQ2.5/DQ8	DQ2.5-glia-γ4c/DQ8-glia-γ1a
	549,273 (+2)	**QQPQQPFPQ**	γ-gliadin/ω-gliadin	1	QQPQQPFPQ (472)	DQ2.5/DQ8	DQ2.5-glia-γ4c/DQ8-glia-γ1a
	597,945 (+3)	P**QQPQQPYPQQPQQP**	γ-gliadin	3	QQPQQPYPQ (458)	DQ2.5/DQ8	DQ2.5-glia-γ3/DQ8-glia-γ1b
					QQPYPQQPQ (464)	DQ2 (glia-γVIb)	—
					PYPQQPQQP (468)	DQ2 (DQ2.2 and DQ2.5)	—
**2**	597,799 (+2)	P**QQPQQPFPQ**	γ-gliadin/ω-gliadin	1	QQPQQPFPQ (472)	DQ2.5/DQ8	DQ2.5-glia-γ4c/DQ8-glia-γ1a
	1198,583 (+1)	T**QQPQQPFPQ**	γ-gliadin	1	QQPQQPFPQ (472)	DQ2.5/DQ8	DQ2.5-glia-γ4c/DQ8-glia-γ1a
	549,273 (+2)	**QQPQQPFPQ**	γ-gliadin/ω-gliadin	1	QQPQQPFPQ (472)	DQ2.5/DQ8	DQ2.5-glia-γ4c/DQ8-glia-γ1a
**3**	1198,585 (+1)	T**QQPQQPFPQ**	γ-gliadin	1	QQPQQPFPQ (472)	DQ2.5/DQ8	DQ2.5-glia-γ4c/DQ8-glia-γ1a
	597,799 (+2)	P**QQPQQPFPQ**	γ-gliadin/ω-gliadin	1	QQPQQPFPQ (472)	DQ2.5/DQ8	DQ2.5-glia-γ4c/DQ8-glia-γ1a
	541,936 (+3)	QQP**PFSQQQQPV**LP	LMW glutenin/γ-gliadin	1	PFSQQQQPV (706)	DQ2.2	DQ2.2-glut-L1
	597,800 (+2)	**PQPQQQFPQ**P	γ-gliadin	1	PQPQQQFPQ (577)	DQ2.5	DQ2.5-glia-γ4b
**4**	597,799 (+2)	P**QQPQQPFPQ**	γ-gliadin/ω-gliadin	1	QQPQQPFPQ (472)	DQ2.5/DQ8	DQ2.5-glia-γ4c/DQ8-glia-γ1a
	1198,586 (+1)	T**QQPQQPFPQ**	γ-gliadin	1	QQPQQPFPQ (472)	DQ2.5/DQ8	DQ2.5-glia-γ4c/DQ8-glia-γ1a
	549,273 (+2)	**QQPQQPFPQ**	γ-gliadin/ω-gliadin	1	QQPQQPFPQ (472)	DQ2.5/DQ8	DQ2.5-glia-γ4c/DQ8-glia-γ1a
	641,983 (+3)	QPQP**FRPQQPYPQ**SQP	α/β-gliadin	1	FRPQQPYPQ (93)	DQ2.5	DQ2.5-glia-α3
	541,936 (+3)	QQP**PFSQQQQPV**LP	LMW glutenin/γ-gliadin	1	PFSQQQQPV (706)	DQ2.2	DQ2.2-glut-L1
**5**	710,354 (+2)	P**QQPQQPFPQ**PQ	γ-gliadin/ω-gliadin	1	QQPQQPFPQ (472)	DQ2.5/DQ8	DQ2.5-glia-γ4c/DQ8-glia-γ1a
	597,799 (+2)	P**QQPQQPFPQ**	γ-gliadin/ω-gliadin	1	QQPQQPFPQ (472)	DQ2.5/DQ8	DQ2.5-glia-γ4c/DQ8-glia-γ1a
	541,936 (+3)	QQP**PFSQQQQPV**LP	LMW glutenin/γ-gliadin	1	PFSQQQQPV (706)	DQ2.2	DQ2.2-glut-L1

Interestingly, the number immune-active peptides detected in all the five genotypes under investigation was lower than in the reference semolina. In particular, none of the immune-stimulatory peptides derived from α-gliadin was detected in genotypes 1, 2, 3 and 5, while only one peptide containing the core epitope DQ2.5-glia-α3 was detected in genotype 4. On the contrary, peptides matching the epitope DQ2.5-glia-γ4c/DQ8-glia-γ1a were detected in all the five genotypes as conserved sequences and two additional core epitopes DQ2.5-glia-γ3/DQ8-glia-γ1b and DQ2.5-glia-γ4b, not reported for semolina digests, were detected in genotype 1 and 3, respectively.

The presence of few intact epitopes (with only one belonging to α/β-gliadins) was a quite promising result that suggested several speculations and paved the way to further detailed investigations. It is feasible that the selected lines contained the main known epitopes at a concentration level, which falls below the method sensitivity, meaning that they could actually be less toxic than commercial semolina. In addition, it is also possible that the selected lines expressed gliadin variants characterized by amino-acids mismatches within the 9 AA core epitopes which induced an higher susceptibility of the intact proteins to the proteolytic activity of digestive enzymes. The latter would result in the hydrolysis of potential epitopes down to shorter fragments (length <9AA, below the minimum peptide binding register) losing their potential to stimulate the T-cell mediated response. This hypothesis was actually supported by the evidence that browsing the hydrolyzed peptide list (identified peptides <9AA in length) some epitopes fragments were detected. For example in line 1, lacking in known full-length α/β-gliadins epitopes, we detected the shorter peptides PL*PYPQP* and *PQLPYPQ*, belonging to the known epitopes DQ2.5-glia- α1b and DQ2.5-glia- α2, respectively.

However, we should also add that a further explanation of our main findings could be the presence of ‘unknown epitopes variants’ that are either not sequenced yet (gliadins full-length sequences not available), or not tested for their toxicity (no confirmation available as binding HLA DQ 2 or DQ8, or as stimulating CD T-cell proliferation or toxicity using human samples or subjects). This would mean that the selected lines could still have high toxicity levels, but the current knowledge did not allow us to draw conclusions about their toxicity risk, and further in-depth investigations would need.

As for this last point, since it is well established that T-cells might also respond to peptides with one or more AA substitutions, we performed also a check for partial sequences matches with known epitopes. The minimum number of identical AA able to raise hazard concerns for celiac patients is challenging to define, because the binding ability to CD-specific MHC molecules and the interaction with T-cells is highly dependent on the nature and position of certain AA^23^. The list of epitopes currently identified discloses a characteristic Q-X_1_-P-X_2_ motif in the large majority of HLA-DQ2 epitopes, and typical motif with glutamine (Q) in position P1 and P9, in the HLA-DQ8 epitopes^23^. In addition, case-by-case *distinguo* based on the position and the nature of adjacent AA sequences is required when performing risk assessment (see Figure 4 point 4.2 and the methods section for details). The number of concerning sequences detected within the list of GDD resistant peptides was summarized in Table 4 confirming the previous trend. The full list can be found in the Supplementary Information (Table S1). In this list, some peptides belonging to α/β-gliadins were identified as potential epitopes variants. For example, the peptide QPQP***F***PA***QQPYPQ***P found in line 1 not only contained the motif QX_1_PX_2_, but also presented a very similar sequence to the known epitope DQ2.5-glia-α3 (only 2 AA mismatches). Still, the actual behavior of these peptides as immune stimulatory epitopes needs to be tested by *in-vitro* T-cell activation tests.

The systematic characterization carried out on a collection of 38 wheat accessions confirmed that durum wheat breeding programs accomplished in the last 25 years improved the pasta-making quality (gluten strength), without causing an increment of toxic epitopes towards CD patients. Tracking the fate of gluten proteins upon *in*-*vitro* simulated gastroduodenal digestion experiments and in-silico toxicity risk assessment confirmed such statement. The selected genotypes boasting medium and strong gluten strength, all presented a significantly lower number of toxic epitopes compared to commercial semolina. Even if none of the five investigated genotypes can be considered safe for CD patients, a lower toxicity level could be envisaged for all of them, and further investigations are required to confirm the potential of the selected lines as valid option for new breeding practices.

Aware of the high heterogeneity of the literature on this topic sometimes reporting contrasting conclusions, here we preferred to follow a very conservative approach for toxicity level prediction with specific reference to guidelines issued by official authorities. However, at this stage we also overestimate the toxicity risk because we neglected the intestinal phase involving brush border proteases in GDD experiments, which was demonstrated to play a key role into final degradation of immune stimulatory peptides^26^. This issue will be addressed in future developments of the current study together with an *in-vitro* immunological evaluation of potential toxicity by T-cell activation experiments.

In perspective, the selected genotypes combining reduced gluten content with conserved rheological properties could represent convenient bases for breeding practices and for the development of new detoxification strategies^69^, decreasing the need for additives to improve dough quality and dietary values of the derived products.

## Methods

### Plant Materials

The 41 genotypes comprised both “cultivated” and “non-cultivated” genotypes, detailed in Table 1 and classified as follows: two accessions of *Triticum aestivum*, one accession of *Triticum monococcum* and 38 accessions of *Triticum turgidum* belonging to six subspecies: *ssp. durum* (26), *ssp. turanicum* (3), *ssp. turgidum* (4), *ssp. polonicum* (1), *ssp. dicoccum* (1) and *ssp. dicoccoides* (3).

The genotypes were grown in the experimental field “A. Martucci” of the Department of Soil, Plant and Food Sciences at Valenzano (Bari, Italy) in 2015, in a randomized complete block design with three field replicates and plots consisting of 1-m rows, 30 cm apart, with 50 germinating seeds per plot. During the growing season, 120 kg ha-1 N were applied and standard cultivation practices were adopted. Plots were hand-harvested at maturity. A seed sample (15 g) per plot was used to determine the thousand-kernel weight (KW).

Harvested grain samples from each plot were separately milled to whole meal on a laboratory mill equipped with 1-mm sieve (Cyclotec Sample Mill, Tecator Foss, Hillerød, Denmark) and stored at +4 °C. The commercial durum wheat semolina used as reference was purchased directly from a local market.

### Collection characterization

#### R5-sandwich ELISA

The gliadin/gluten content of wheat flours was determined with a commercial kit based on R5 monoclonal antibody sandwich assay (RIDASCREEN® Gliadin Assay R7001, R-Biopharm) developed for the quantitative analysis of prolamins from wheat (gliadin), rye (secalin) and barley (hordein). Two independent extractions were carried out for each genotypes (25 mg of flour) with patented Cocktail solution developed by E. Mendez (patent WO 02/092633, R7006, R-Biopharm). The assay was carried out according to the provider instruction, and each sample was tested on two different wells. The absorbance at 450 nm was measured by a 96-well microplate reader (Varioskan Flash, Thermo Fischer Scientific). The R5 reactivity for each sample was quantified by interpolation of the calibration curves calculated with gliadin standard solutions provided within the kit. The absolute gliadin concentration for each genotype was divided by the semolina averaged value in order to evaluate the percent relative variation in R5 reactivity compared to our reference.

#### Sequential gliadin extraction and HPLC-UV analysis

Gliadins extraction was carried out as previously described^70^, with few modifications. Non-defatted flours (100 mg) were extracted with a buffered salt solution (2 × 1 mL 0.067 M phosphate buffer, 0.4 M NaCl, pH 7.6) at room temperature to separate albumins and globulins. The pellet was extracted with alcoholic solution (3 × 0.5 mL 60% ethanol solution) at room temperature to collect gliadins fraction. After the addition of each extraction solution, the suspensions were vortexed for 2 min, sonicated in a water bath for 5 min and shaked for 10 min. The three supernatants collected after centrifugation (10 min at 5000 rcf) were combined and further centrifuged for 10 min at 5000 rcf. This samples were stored at room temperature and analysed within 12 hours.

The analysis was carried out on a fully automated on-line SPE (solid phase extraction) HPLC-UV-MS system consisting in a Ultimate 3000 UHPLC provided with dual pump, an auto-sampler equipped with a 10-port switching valve and a diode array detector (Thermo Fisher Scientific, San Jose, USA). The gliadin extracts were diluted 1:10 in 0.1% trifluoroacetic acid (TFA) aqueous solution and a 50 µL aliquot of sample was injected. The sample was purified by means of Acclaim™ 300 C-18 cartridges (2 × 10 mm, 3 µm, 300 Å, Thermo Fisher Scientific). The purified protein pool was automatically recovered from the cartridge and injected into an Aeris WIDEPORE XB-C8 column (250 × 2.10 mm, 3.6 µm, 200 Å, Phenomenex) for the chromatographic separation. The chromatographic conditions were set as follows: solvent A, 0.1% TFA in water; solvent B 0.1% TFA in acetonitrile; gradient: 0–7 min in isocratic 25% B, 7–22 min linear 25–31% B, 22–59 min linear 31–40%B, 59–82 min linear 40–47% B; 82 min step change to 90% B, 82–97 isocratic 90% B, 97 step change to 25%B, 97–117 isocratic 25% B, flow rate 0.25 mL/min, column temperature 55 °C. Detection of UV absorbance was carried out at 210 nm. Each UV chromatogram was investigate for peak integration and quantitation of gliadins classes. The peak attribution was as follows: t_R_ 25–35 min ω-gliadins, 35–52 min α/β-gliadins, 52–75 min γ-gliadins. Three extraction replicates were performed for each genotype.

#### Grains quality characterization

The collection has been characterized for grain yield per spike (GYS) defined as the product of kernel number per spike and one-thousand kernel weight, these components being in direct connection with productivity in wheat^71–75^. The grain protein content (GPC), was assessed on 3 g of whole meal flour using a dual beam near infrared reflectance spectrophotometer (Zeutec Spectra Alyzer Premium, Zeutec Büchi, Rendsburg, Germany).

Wet gluten (WG) and gluten index (GI) were determined on 10 g of milled grain samples according to the ICC standard No. 155 (ICC 1994) by means of the complete system consisting of glutomatic 2200, Centrifuge 2015, and Glutork 2020 (Perten Instruments AB, Huddinge, Sweden). First, WG was evaluated, and GI was calculated as the percent ratio of the WG fraction remaining on the sieve after centrifugation (Centrifuge 2015, Perten Instruments AB, Huddinge, Sweden) to the total WG weight. Dry gluten (DG) was determined by drying the total WG at 150 °C for 4 min by means of Glutork 2020 apparatus (Perten Instruments 0AB, Huddinge, Sweden). Gluten hydration index (GHI) was calculated as the percent ratio (WG − DG)/WG. Results were the average of two analytical replications, as previously reported.

#### Statistical data processing

Statistically significant differences in R5-ELISA reactivity and gliadins content (HPLC-UV) between commercial semolina and each genotype under investigation were determined by multiple t-tests comparing mean values from small samples at a significance level of 5% (hypothesis case: comparison of small samples with unknown but equal variances, the latter assumption was taken after a proper F-test for equality of two variances,). All further statistical data analysis was carried out by Statistica 7.0 software. Pearson’s product moment correlations were calculated between contents of α/β-gliadins, γ-gliadins, ω-gliadins, total gliadins, their percentage amounts, R5-reactivity, grain protein content, grain yield per spike, dry gluten and gluten index for all analysed genotypes (41 cases). PCA with NIPALS algorithm was carried out (12 variables, 42 cases, fitting method: number of components by v-fold cross-validation, v = 7) to determine if the variables under investigation could be used to differentiate between cultivated (C) and non-cultivated (NC) wheat genotypes. In addition, hierarchical cluster analysis by joining tree was performed on original variables setting Ward’s methods amalgamation rule and selecting Euclidean distances; only the HPLC-UV peak areas were scaled down (factor 1E5) to avoid over-weighting of this variable on the cluster calculation.

### Evaluation of gastroduodenal digestibility and potential toxicity of selected genotypes

#### *In vitro*-simulation of human gastroduodenal digestion

Selected wheat flours (1 g) were subjected to *in-vitro* simulated human gastroduodenal (GD) digestion by standardized static model proposed by Minekus *et al*.^54^. Simulated salivary fluid (SSF), simulated gastric fluid (SGF), and simulated intestinal fluid (SIF) (without phospholipids) were prepared according to the harmonized conditions. As for duodenal phase bile salts and pancreatin were used, after proper evaluation of the trypsin activity by TAME assay^76^. The reaction was stopped by addition of protease inhibitor (phenylmethylsulfonyl fluoride) and the resulting digests were store at −20 °C until further analysis.

#### Micro-HPLC-MS/MS analysis and peptide identification

GD digests were purified by Sep-Pak C18 cartridges (50 mg, 1 mL, Waters spa, Milano, Italy). Each column was used as follows: (i) conditioning/equilibration step (3 × 1 mL methanol, 3 × 1 mL SIF), (ii) sample loading (1 mL digest), (iii) washing step (1 × 0.5 mL water), (iv) elution step (1 × 1 mL 0.1% formic acid in H_2_O:CH_3_CN 10:90). Finally, the eluates were 0.2 µm-filtered before MS analysis.

Micro-HPLC-MS/MS analyses were performed on an Ultimate 3000 UHPLC system coupled to a hybrid quadrupole-Orbitrap^TM^ mass spectrometer Q-Exactive Plus (Thermo Fisher Scientific, San Josè, USA). Peptide separation was accomplished on an Acclaim PepMap100, C18 column, (3 μm, 100 Å, 1 × 150 mm) at a flow rate of 60 µL/min, using a binary gradient under the following conditions: solvent A 0.1% formic acid in H_2_O, solvent B 0.1% formic acid in CH_3_CN/ H_2_O 80:20, multistep-gradient: 0–50 min linear 12–45% B, 50 min step change to 50% B, 50–60 min isocratic at 50% B, 60 min step change to 90% B, 60–75 min isocratic at 90%, 75 min step change to 12% B, 75–95 min isocratic at 12% B, column temperature 25 °C, injection volume 2 µL.

Untargeted high resolution MS/MS analysis was performed by Full-MS/dd-MS^2^ analisis mode set up as follows: Full-MS: microscan 1, resolution 70 k, AGC target 1e6, maximum injection time 30 ms, lscan range 250–3500 m/z; dd-MS^2^ microscan 1, resolution 17.5 k, AGC target 5e4, maximum injection time 50 ms, loop count 10, isolation window 2.0 m/z, stepped collision energy 27, 30, minimum AGC target 2.5e2, charge exclusion unassigned, >8, peptide match preferred, exclude isotopes on, dynamic exclusion 20 s. Raw data were processed by Proteome Discoverer 2.1 (Thermo Fisher Scientific) for peptide/protein identification. The Sequest HT searching algorithm against a curated open-source wheat gluten protein sequence database (GluPro V1.0) was selected for experimental spectra matching^64^. The processing workflow was set as follows: unspecific cleavage, mass tolerance on the precursor and fragment ions 10 ppm and 0.02 Da, respectively, peptide length 5–144 amino acids (AA), dynamic modification methionine-oxidation, glutamine-deamidation. Specific filters on the software output were applied in order to constrain the peptide list to the most reliable sequence identifications: peptide-spectrum matches (PSM) only unambiguous, number of PSM ≥ 3, mass tolerance on the assigned precursor ion 5 ppm, confidence level at least medium (FDR ≤ 5%).

#### In-silico evaluation of potential toxicity

The in-silico evaluation of the risk for toxicity of the selected genotypes was carried out according to the guidelines provided by EFSA in 2017^23^. First the peptide list was refined with the size cut-off of 9 AA (peptide binding register^65^) to only stable gastro-duodenal resistant peptides; then 100% identity matches with known T-cell stimulatory epitopes was highlighted by means of the CD Novel Protein Risk Assessment Tool provided as open-source by the FARRP at University of Nebraska, (CELIAC Database, Beta-3 Release^66^); finally, search for partial sequence identity was performed as follow: (i) the QX_1_PX_2_ motif should be placed at position P4 or P6 of the 9AA core epitope, in order to act as affinity target for the enzyme tissue transglutaminase 2, (ii) the AA occurrence in position X_1_ and X_2_ was restricted to certain AA (X_1_ = L, Q, F, S, and X_2_ = Y, F, A, V, Q), (iii) few known CD peptide sequences do not contain the QX_1_PX_2_ motif, therefore the partial match with such epitopes should additionally be considered allowing a maximum of 3 AA mismatches. These and other practical considerations provided by EFSA, for partial sequence identities with likelihood to activate immune response were used for peptide screening.

## Electronic supplementary material

supplementary information

## Data Availability

All data generated or analyzed during the study are included in the article and in the Supplementary Information.
