# Supplementary material for: Scouting for Naturally Low-Toxicity Wheat Genotypes by a Multidisciplinary Approach
Source: Sci Rep. 2019 Feb 7;9:1646. doi: 10.1038/s41598-018-36845-8 (PMC6367382; doi:10.1038/s41598-018-36845-8)
Supplement: Supplementary file 1 — supplementary information [file 41598_2018_36845_MOESM1_ESM.docx]

**Supplementary Information of**

**SCOUTING FOR NATURALLY LOW-TOXICITY WHEAT GENOTYPES BY A MULTIDISCIPLINARY APPROACH**

**Rosa Pilolli^1^*, Agata Gadaleta^1,2^, Gianfranco Mamone^3^,**

**Domenica Nigro^4^, Elisabetta De Angelis^1^, Nicola Montemurro^1^, Linda Monaci^1^**

*^1^ Institute of Sciences of Food Production, National Research Council of Italy (ISPA-CNR), via Giovanni Amendola 122/O - 70126 Bari, Italy;*

*^2^ Department of Agricultural & Environmental Sciences, Università degli Studi di Bari Aldo Moro, via G. Amendola 165/A – 70126 Bari, Italy;*

*^3^* *Institute of Food Sciences, National Research Council of Italy (ISA-CNR), via Roma, 64 -83100 Avellino, Italy;*

*^4^ Department of Soil, Plant & Food Sciences, Università degli Studi di Bari Aldo Moro, via G. Amendola 165/A – 70126 Bari, Italy.*

* Corresponding author:

*rosa.pilolli@ispa.cnr.it*

**Supporting Information**

**Table S1.** Summary of the concerning GD resistant peptides containing partial matches with known T-cell stimulatory epitopes. The QX_1_QX_2_ motifs were highlighted with bold font style. Potential epitopes variants with maximum 3 AA mismatches were highlighted with italic font style and the mismatches were underlined.

| ***Sequence*** | ***Protein*** | ***XCorr Sequest HT*** | ***m/z [Da] (z)*** | ***ΔM [ppm]*** | ***Sequence Length*** | ***# PSMs*** |
| --- | --- | --- | --- | --- | --- | --- |
| ***Sample REF4: Commercial semolina*** | | | | | | |
| QPQQPFPQT**QQPQQPF** | γ gliadin | 2,45 | 641,983 (+3) | 0,06 | 16 | 3 |
| QFPGQ**QQPF**PPQQP | α/β gliadin | 2,71 | 812,397 (+2) | -2,55 | 14 | 9 |
| PQQS**QQPF**PQQP | ω gliadin | 2,74 | 1409,676(+1) | -3,48 | 12 | 11 |
| PQQIFP**QQPQ** | γ gliadin | 1,95 | 605,815 (+2) | 0,31 | 10 | 4 |
| PQLPFP**QQPQ** | γ gliadin | 2,65 | 1179,618(+1) | 1,58 | 10 | 14 |
| PQQS**QQPF**PQ | ω gliadin | 2,09 | 592,789 (+2) | 0,24 | 10 | 3 |
| GIIQP**QQPA** | γ gliadin | 1,8 | 951,525 (+1) | -1,01 | 9 | 5 |
| PQQ**QFPQ**PQ | γ gliadin | 2,19 | 549,273 (+2) | 0,75 | 9 | 17 |
| QQPPFSQ**QQPF**PQQPP | LMW glutenin | 1,16 | 940,456 (+2) | 4,05 | 16 | 17 |
| QPQQPQRPFPQS**QQPQ** | γ gliadin | 1,09 | 640,652 (+3) | 2,59 | 16 | 5 |
| QPQQPFPQT**QQPQ**QP | γ gliadin | 2 | 592,960 (+3) | -0,38 | 15 | 3 |
| PQQPQLPSP**QQPQ**QP | γ gliadin | 1,41 | 566,950 (+3) | -4,6 | 15 | 6 |
| QPQPFPA**QQPY**PQP | α/β gliadin | 1,85 | 812,396 (+2) | 2,87 | 14 | 32 |
| QPPFSQQQ**QQPV**LP | LMW glutenin | 1,42 | 541,937 (+3) | -3,88 | 14 | 8 |
| QTFP**QQPQ**LPFPQ | γ gliadin | 1,53 | 519,269 (+3) | 0,6 | 13 | 3 |
| QPFPQS**QQPQ**QP | γ gliadin | 1,53 | 705,344 (+2) | 0,19 | 12 | 5 |
| PQQS**QQPF**TQPQ | ω gliadin | 1,26 | 707,343 (+2) | 1,46 | 12 | 3 |
| QPQQ**QFPQ**TQQP | γ gliadin | 2,01 | 486,555 (+3) | -2,98 | 12 | 7 |
| QQS**QQPF**TQPQQ | ω gliadin | 1,44 | 483,215 (+3) | -2,61 | 12 | 3 |
| QQPIP**QQPQ**QP | ω gliadin | 1,72 | 644,835 (+2) | -0,62 | 11 | 4 |
| QQPFC**QQPQ**Q | γ gliadin | 1,7 | 411,517 (+3) | -0,08 | 10 | 9 |
| QTFP**QQPQ**LP | γ gliadin | 1,36 | 592,309 (+2) | 0,35 | 10 | 3 |
| PQLPFP**QQPQ** | γ gliadin | 1,3 | 590,803 (+2) | -0,86 | 10 | 3 |
| AQLPFP**QQPQ** | γ gliadin | 1,67 | 577,304 (+2) | 0,84 | 10 | 7 |
| PQP**QQPQ**QP | γ gliadin / ω-gliadin | 1,23 | 524,264 (+2) | -0,94 | 9 | 3 |
| QFPQQ**QFPQ** | ω gliadin | 0,98 | 574,283 (+2) | 4,2 | 9 | 3 |
| QHP**QQPF**PQ | γ gliadin | 1,7 | 553,772 (+2) | -0,22 | 9 | 3 |
| PQQP**QLPF**PQ | γ gliadin | 2,46 | 590,312 (+2) | 1,11 | 10 | 14 |
| QHQ**QLPQ**PQ | γ gliadin | 1,16 | 552,776 (+2) | 1,56 | 9 | 8 |
| PQQ**QLPQ**PQ | γ gliadin | 1,24 | 532,281 (+2) | 0,86 | 9 | 4 |
| PQLPYPQPQPFP*PQQSYPQPQ* | α/β gliadin | 3,84 | 1231,614(+2) | 2,67 | 21 | 16 |
| QQ*PQQPLPQPQ* | γ gliadin | 2,53 | 644,835 (+2) | -0,81 | 11 | 19 |
| Q*PQQAFPQQP* | γ gliadin / ω-gliadin | 2,25 | 584,791 (+2) | -0,03 | 10 | 4 |
| R*PQQPYPQPQ* | α/β gliadin | 2,05 | 619,817 (+2) | -0,52 | 10 | 4 |
| *PQQPFLQPQ* | γ gliadin / ω-gliadin | 1,56 | 1082,563(+1) | 0,51 | 9 | 6 |
| *PQQPFPQPQ* | γ gliadin / ω-gliadin / α-gliadin | 2,42 | 1066,531(+1) | -0,58 | 9 | 17 |
| *PQQPYPQPQ* | α/β gliadin | 2,19 | 541,767 (+2) | 0 | 9 | 9 |
| *PQQPYPQPQ*P | α/β gliadin | 1,61 | 590,294 (+2) | 0,39 | 10 | 20 |
| Q*PQQQVPQPQ* | γ gliadin | 1,02 | 589,301 (+2) | -1,67 | 10 | 4 |
| *PQLPFPQPQ* | γ gliadin | 1,62 | 526,283 (+2) | 0,88 | 9 | 6 |
| ***Sample 1: Duetto (T. turgidum ssp. durum)*** | | | | | | |
| PQLPFP**QQPQ** | γ gliadin | 2,07 | 590,312 (+2) | 0,07 | 10 | 4 |
| SQQ**QQPV**IPQ | LMW glutenin / γ-gliadin | 2,29 | 576,804 (+2) | 0,52 | 10 | 10 |
| PQQS**QQPF**PQ | ω-gliadin | 1,25 | 1184,567(+1) | -1,96 | 10 | 3 |
| QPQPFPA**QQPY**PQP | α/β gliadin | 1,57 | 812,398 (+2) | 4,82 | 14 | 8 |
| QPQQ**QFPQ**PQ | γ gliadin | 1,39 | 613,301 (+2) | -1,61 | 10 | 6 |
| PQQ**QFPQ**PQ | γ gliadin | 1,75 | 549,273 (+2) | 0,3 | 9 | 7 |
| PQQPF**QQPQ** | ω-gliadin | 1,77 | 549,272 (+2) | -0,47 | 9 | 4 |
| QHP**QQPF**PQ | γ gliadin | 1,22 | 553,772 (+2) | -0,77 | 9 | 3 |
| PQQP**QLPF**PQ | γ gliadin | 2,32 | 590,312 (+2) | 0,28 | 10 | 16 |
| Q*PQQIFPQPQ* | γ gliadin | 1,91 | 605,815 (+2) | 1,42 | 10 | 6 |
| *PQQPFPQPQ* | γ gliadin / ω-gliadin / α-gliadin | 2 | 533,770 (+2) | 0,38 | 9 | 11 |
| *QQQPFPQPQ* | γ gliadin | 2,41 | 1097,537(+1) | -0,3 | 9 | 12 |
| Q*PQQAFPQQP* | γ gliadin / ω-gliadin | 1,68 | 584,791 (+2) | -0,35 | 10 | 3 |
| *PQQPYPQPQ*P | α/β gliadin | 1,85 | 590,293 (+2) | -0,75 | 10 | 21 |
| *PQLPFPQPQ* | γ gliadin | 1,65 | 526,283 (+2) | 1,23 | 9 | 6 |
| *PQQPYPQPQ* | α/β gliadin | 1,47 | 541,767 (+2) | 1,02 | 9 | 5 |
| ***Sample 2: Colosseo (T. turgidum ssp. durum)*** | | | | | | |
| PQQ**QFPQ**PQ | γ gliadin | 2,27 | 549,272 (+2) | 0,08 | 9 | 6 |
| QPPF*SQQQ****QQPV***LP | LMW glutenin | 1,1 | 541,936 (+3) | -4,78 | 14 | 4 |
| PQQS**QQPF**PQQP | ω gliadin | 0,99 | 1409,675(+1) | -4,08 | 12 | 3 |
| PQQS**QQPF**TQPQ | ω gliadin | 1,24 | 707,343 (+2) | 1,81 | 12 | 3 |
| QTFP**QQPQ**LP | γ gliadin | 1,37 | 592,309 (+2) | 0,24 | 10 | 4 |
| SQQ**QQPV**LPQ | LMW glutenin / γ-gliadin | 1,89 | 576,804 (+2) | 0,1 | 10 | 6 |
| QFPQQ**QFPQ** | ω gliadin | 1,63 | 574,281 (+2) | 1,01 | 9 | 3 |
| GIIQP**QQPA** | γ gliadin | 1,13 | 476,267 (+2) | 0,32 | 9 | 3 |
| QHP**QQPF**PQ | γ gliadin | 1,86 | 553,772 (+2) | -0,22 | 9 | 3 |
| PQQP**QLPF**PQ | γ gliadin | 2,47 | 590,312 (+2) | 1 | 10 | 16 |
| PQQP**QLPF**PQP | γ gliadin | 0,95 | 638,835 (+2) | -4,07 | 11 | 3 |
| Q*PQQIFPQPQ* | γ gliadin | 2,36 | 605,815 (+2) | 0,71 | 10 | 9 |
| Q*PQQAFPQQP* | γ gliadin / ω-gliadin | 2,21 | 584,791 (+2) | 0,28 | 10 | 3 |
| *QQQPFPQPQ* | γ gliadin | 2,26 | 549,272 (+2) | 0,08 | 9 | 8 |
| *PQQPFPQPQ* | γ gliadin / ω-gliadin / α-gliadin | 2,29 | 533,770 (+2) | 0,61 | 9 | 16 |
| Q*PQQTFPQPQ* | γ gliadin | 1,8 | 599,797 (+2) | 0,83 | 10 | 5 |
| *PQQPYPQPQ* | α/β gliadin | 1,17 | 541,767 (+2) | 0,9 | 9 | 5 |
| *QQQPQPFPQ* | LMW glutenin | 2,03 | 1097,534(+1) | -3,41 | 9 | 6 |
| *QQPQQAFPQ*P | γ gliadin | 1,18 | 584,792 (+2) | 1,64 | 10 | 3 |
| ***Sample 3: Lloyd (T. turgidum ssp. durum)*** | | | | | | |
| SQQ**QQPV**LPQ | LMW glutenin / γ-gliadin | 1,99 | 576,804 (+2) | 0,63 | 10 | 14 |
| PQLPFP**QQPQ** | γ gliadin | 2,35 | 590,312 (+2) | 0,49 | 10 | 6 |
| PQQ**QFPQ**PQ | γ gliadin | 2,02 | 549,272 (+2) | 0,19 | 9 | 11 |
| QPQPFPA**QQPY**PQP | α/β gliadin | 1,63 | 812,397 (+2) | 3,99 | 14 | 4 |
| QPPFSQQQ**QQPV**LP | LMW glutenin | 1,18 | 541,937 (+3) | -3,76 | 14 | 5 |
| QQS**QQPF**TQPQQ | ω-gliadin | 1,11 | 483,215 (+3) | -2,3 | 12 | 3 |
| QPQQ**QFPQ**TQQP | γ gliadin | 1,41 | 486,555 (+3) | -2,29 | 12 | 4 |
| AQLPFP**QQPQ** | γ gliadin | 1,62 | 577,304 (+2) | 0,84 | 10 | 5 |
| QQFPQT**QQPQ** | γ gliadin | 1,23 | 616,772 (+2) | -4,92 | 10 | 3 |
| QHP**QQPF**PQ | γ gliadin | 1,24 | 553,773 (+2) | 0 | 9 | 3 |
| QQPIP**QQPQ** | ω-gliadin | 1,78 | 532,281 (+2) | 0,63 | 9 | 8 |
| PQQP**QLPF**PQ | γ gliadin | 2,79 | 590,312 (+2) | 0,38 | 10 | 16 |
| QQ*PQQPLPQPQ* | γ gliadin | 2,2 | 644,836 (+2) | 0,13 | 11 | 14 |
| Q*PQQPFPQPQ* | γ gliadin / ω-gliadin / α-gliadin | 3,28 | 1194,591(+1) | 0,83 | 10 | 20 |
| *PQQPFPQPQ* | γ gliadin / ω-gliadin / α-gliadin | 2,13 | 533,770 (+2) | 0,27 | 9 | 11 |
| *QQQPFPQPQ* | γ gliadin | 1,57 | 1097,534(+1) | -2,74 | 9 | 7 |
| *PQQPYPQPQ*P | α/β gliadin | 1,44 | 590,293 (+2) | -0,44 | 10 | 8 |
| *PQQPYPQPQ* | α/β gliadin | 1,49 | 541,767 (+2) | 0,45 | 9 | 6 |
| *QQQPQPFPQ* | LMW glutenin | 1,99 | 549,273 (+2) | 0,53 | 9 | 8 |
| *QQPQQAFPQ*P | γ gliadin | 1,21 | 584,792 (+2) | 2,27 | 10 | 3 |
| ***Sample 4: Neolatino (T. turgidum ssp. durum)*** | | | | | | |
| PQLPFP**QQPQ** | γ gliadin | 2,28 | 590,311 (+2) | -0,34 | 10 | 6 |
| QPQPFPA**QQPY**PQP | α/β gliadin | 1,42 | 812,397 (+2) | 3,92 | 14 | 6 |
| AQLPFP**QQPQ** | γ gliadin | 1,66 | 577,304 (+2) | 0,94 | 10 | 4 |
| QQFPQT**QQPQ** | γ gliadin | 1,16 | 616,773 (+2) | -4,13 | 10 | 6 |
| SQQ**QQPV**LPQ | LMW glutenin | 1,89 | 576,804 (+2) | -0,01 | 10 | 14 |
| QPQQ**QFPQ**PQ | γ gliadin | 1,45 | 613,301 (+2) | -0,51 | 10 | 4 |
| PQQ**QFPQ**PQ | γ gliadin | 1,61 | 549,272 (+2) | 0,08 | 9 | 8 |
| PQQP**QLPF**PQ | γ gliadin | 2,81 | 590,312 (+2) | 1,31 | 10 | 17 |
| Q*PQQPFPQPQ* | γ gliadin / ω-gliadin / α-gliadin | 3,76 | 1194,592(+1) | 1,85 | 10 | 24 |
| *QQQPFPQPQ* | γ gliadin | 2,31 | 1097,533(+1) | -3,74 | 9 | 12 |
| *PQQPFPQPQ* | γ gliadin / ω-gliadin / α-gliadin | 2,23 | 533,770 (+2) | 1,3 | 9 | 16 |
| *PQQPYPQPQ* | α/β gliadin | 1,13 | 541,767 (+2) | 0,79 | 9 | 5 |
| ***Sample 5: PI 56263 (T. turgidum ssp. turgidum)*** | | | | | | |
| QPQPFPA**QQPY**PQP | α/β gliadin | 1,92 | 812,396 (+2) | 3,09 | 14 | 9 |
| QPFPQS**QQPQ**QP | γ gliadin | 1,39 | 1409,684(+1) | 2,59 | 12 | 3 |
| PQQPFPQS**QQPQ** | γ gliadin | 1,41 | 1409,684(+1) | 2,59 | 12 | 4 |
| PQQS**QQPF**PQQP | ω-gliadin | 1,46 | 1409,684(+1) | 2,59 | 12 | 6 |
| AQLPFP**QQPQ** | γ gliadin | 1,97 | 577,304 (+2) | -0,22 | 10 | 5 |
| PQLPFP**QQPQ** | γ gliadin | 2,28 | 590,312 (+2) | 0,28 | 10 | 9 |
| QPFPQP**QQPQ** | γ gliadin | 2,33 | 597,799 (+2) | 0,77 | 10 | 4 |
| QPPFSQQQ**QQPV**LP | LMW glutenin | 1,17 | 541,936 (+3) | -4,89 | 14 | 10 |
| SQQ**QQPV**LPQ | LMW glutenin / γ-gliadin | 1,59 | 576,805 (+2) | 0,84 | 10 | 4 |
| QQQIP**QQPQ** | ω-gliadin | 1,74 | 547,784 (+2) | 1,1 | 9 | 3 |
| QQPIP**QQPQ** | ω-gliadin | 1,18 | 532,281 (+2) | 0,63 | 9 | 6 |
| QQPQQ**QFPQ** | γ gliadin | 0,91 | 564,775 (+2) | 0,01 | 9 | 4 |
| PQQ**QFPQ**PQ | γ gliadin | 1,77 | 549,273 (+2) | 0,97 | 9 | 7 |
| PQQP**QLPF**PQ | γ gliadin | 2,9 | 590,312 (+2) | 0,28 | 10 | 14 |
| PQQP**QLPF**PQP | γ gliadin | 0,87 | 638,837 (+2) | -1,39 | 11 | 3 |
| *PQQPFPQPQ* | γ gliadin / ω-gliadin / α-gliadin | 2,38 | 533,770 (+2) | 0,73 | 9 | 11 |
| *PQQPFLQPQ* | γ gliadin / ω-gliadin | 2,09 | 541,786 (+2) | 0,9 | 9 | 3 |
| *PQQPFPLQP*Q | ω-gliadin | 1,06 | 590,314 (+2) | 3,8 | 10 | 3 |
| *PQQPYPQPQ* | α/β gliadin | 1,43 | 541,767 (+2) | 0,11 | 9 | 7 |

**Figure S1.** Paired scatterplots between the R5-ELISA reactivity and the HPLC-UV analyses.
